# Phytochemistry, Pharmacology and Toxicology of *Spilanthes acmella*: A Review

**DOI:** 10.1155/2013/423750

**Published:** 2013-11-26

**Authors:** Suchita Dubey, Siddhartha Maity, Mahendra Singh, Shubhini A. Saraf, Sudipta Saha

**Affiliations:** ^1^Department of Pharmaceutical Sciences, School of Bioscience and Biotechnology, Babasaheb Bhimrao Ambedkar University, Lucknow, Uttar Pradesh 226025, India; ^2^Department of Pharmaceutical Technology, Jadavpur University, Kolkata, West Bengal 700032, India

## Abstract

*Spilanthes acmella* is an important medicinal plant, found in tropical and subtropical countries mainly India and South America. Popularly, it is known as toothache plant which reduces the pain associated with toothaches and can induce saliva secretion. Various extracts and active metabolites from various parts of this plant possess useful pharmacological activities. Literature survey proposed that it has multiple pharmacological actions, which include antifungal, antipyretic, local anaesthetic, bioinsecticide, anticonvulsant, antioxidant, aphrodisiac, analgesic, pancreatic lipase inhibitor, antimicrobial, antinociception, diuretic, vasorelaxant, anti-human immunodeficiency virus, toothache relieve and anti-inflammatory effects. This review is elaborately describing the traditional uses, phytochemistry, pharmacology, and toxicology of this plant. This review would assist researchers to search scientific information in the future.

## 1. Introduction

The increasing demand on herbal medicines and their acceptance in international market because of potent pharmacological potential and high therapeutic value have been proving to be real blessing to the people. However, efforts are needed to explore, standardise, and validate ayurvedic medicines for their potency, safety, and efficacy in order to bring them to market as main line therapeutics. *Spilanthes acmella* refers to the important medicinal plant distributed in the tropical and subtropical regions around the world with rich source of therapeutic and medicinal constituents. The main constituents, namely, “spilanthol” and “acmellonate”, are sometimes used to reduce the pain associated with toothaches and can induce saliva secretion [1, 2]. Other important traditional uses of this herb are the following: treatment of rheumatism, as a sialagogue for stammering, tongue paralysis, antipyretic, sore throat, and gum infections [3], and as an antipyretie herb. *Spilanthes acmella *has been well documented for its uses as spices, as antiseptic, antibacterial, antifungal, and antimalarial, treatment, and as remedy for toothache, flu, cough, rabies diseases, and tuberculosis [2, 4]. 

## 2. Traditional Uses

This plant is very popular among the ancient tribal community; special food item is prepared from this plant in religious festival. The poor people offered this plant along with the “Ajeng Dues” in Dobur Uie [5]. In particular, this plant is famous as a folklore remedy for toothache and for throat and gum infections [6]. The flowers are crushed and applied at the site of toothache, particularly in “Irula tribe of Hasanur hills in Erode district of Tamilnadu” where it is known by the local name “Mandal Poo Chedi” [7]. Apart from Tamil Nadu, root paste of the plant is used in throat problems in Chindwara and Betul district of Madhya Pradesh [8]. The plant is also known to be used as panacea (Sumatra), as stimulant, for toothache (Sudan), for stomatitis (Java), and for wound healing (India) [9]. In Cameroon, the plant is used as a snakebite remedy and in the treatment of articular rheumatism [10]. It is supposed to be useful in cases of tuberculosis [4]. In India, *S. acemella* flower heads are used to treat stammering in children. Leaves and flowers of the plant are also used to treat leucorrhoea in females among people of tribes in Bangladesh [9]. The whole plant paste of *Spilanthes acmella* is also used as “poisonous sting” in Chittagong hill tracts of Bangladesh where the plant is also known as Jhummosak [11]. 

## 3. Phytochemistry 

It is necessary to explore the phytochemical constituents of any medicinal plant to establish a relation between pharmacology and chemistry of the plant. Many studies have been carried out for chemical analysis and structural determination of pungent alkamides from *Spilanthes acmella*. The major pungent constituent reported in this plant *S. acemella *is “spilanthol,” which is an isobutylamide and is well known for its insecticidal properties [12, 13]. The flower head and root part of the plant have been reported to be the rich source of active principles. Triterpenoids have also been found in the plant [14]. Spilanthol is chemically N-isobutylamide which is bitter in taste and could stimulate salivation. The molecular formula of spilanthol was determined as (2E,6Z,8E)-N-isobutylamide-2,6,8-decatrienamide [15]. Spilanthol has a strong pungent taste; it may produce local astringency and anaesthetic effects. *Spilanthes acmella* contains secondary metabolites which are summarised in Table 1. Spilanthol can be concentrated in the ethanol extract, which has once been found to contain 9.04% of total N-alkylamides yet 88.84% spilanthol [16]. 

## 4. Pharmacology

*Spilanthes acmella* has multiple pharmacological actions which are summarized in Table 2.

### 4.1. Local Anaesthetic Activity [17]

The local anaesthetic activity of *Spilanthes acmella* has been carried out using two different animal models: (i) intracutaneous wheal in guinea pigs using nupercaine as standard (suitable for determining degree of anaesthesia) and (ii) plexus anaesthesia in frog using cocaine as standard (used for determining onset of anaesthesia). The mean onset of local anaesthetic action was very potent which could be attributed to the presence of alkylamides.

### 4.2. Antipyretic Effects [18]

Chakraborty et al. (2010) studied the antipyretic activity of *Spilanthes acmella* which was carried out by yeast induced method as yeast is commonly used for the induction of pyrexia. The dose varies accordingly in various studies. Various workers used different concentrations and different doses of yeast. The antipyretic activity of *Spilanthes acmella* demonstrated in the study is attributed to the presence of flavonoids which are predominant inhibitors of either cyclo-oxygenase or lipo-oxygenase [19]. 

### 4.3. Anti-Inflammatory/Analgesic Activity [20]

 The anti-inflammatory activity of *Spilanthes acmella* has been carried out by the researchers using carrageenan induced hind paw oedema. Carrageenan is a standard phlogistic agent to study anti-inflammatory activity. The extract was found to produce considerable dose-dependent inhibition of paw oedema which was less than the standard drug. They also demonstrated the analgesic activity of *Spilanthes acmella* using acetic acid induced abdominal constriction and tail flick method. The former procedure is often used to evaluate the activity of peripherally acting analgesics while the later indicates the involvement of central nervous system. The aqueous extract produced better results as compared to tail flick method which meant that the plant can be explored as peripherally acting analgesic. The activity was attributed to the presence of flavonoids which are potent inhibitors of prostaglandins at later stages of acute inflammation.

### 4.4. Antifungal Activity [21]

The effect of different concentrations of *Spilanthes acmella* flower head extract against four different fungi: *Aspergillus niger, Aspergillus parasiticus, Fusarium oxysporum,* and *Fusarium moniliformi, *was evaluated by Rani and Murty (2006). All the concentrations of the test solution inhibited the fungal species with varying degree of sensitivity. The maximum zone of inhibition was found to be for highest concentration and increased proportionally with the dose. Among the test organisms, high inhibition zones were observed in *F. oxysporium* and* F. moniliformis* followed by *A. niger* and *A. paraiticus.*

### 4.5. Diuretic Effect

The diuretic potential of *Spilanthes acmella *whole plant as well as freshflowers has been extracted using cold water extract method and shows that the highest dose of flowers tested possesses strong diuretic activity when given orally in a single dose [22, 23]. The diuresis induced by the *Spilanthes acmella *flowers was found to be strong with intensity similar to that of furosemide and accompanied by marked increases in both urinary Na^+^ and K^+^ levels. Researchers proposed that since the urine was slightly acidified, this suggests that it is acting as a loop diuretic. Phytochemically, *Spilanthes acmella *flowers are shown to contain N-isobutylamides [2], alkaloids [24], and amino acids [24, 25]. Therefore, *Spilanthes acmella *flowers may be useful as a nontoxic natural therapeutic agent in the treatment of such conditions by traditional practitioners. The onset of the diuretic action of the aqueous extract was extremely rapid, and it also had a fairly long duration of action. This is an appealing diuretic profile as it would curtail the frequency of administration. 

### 4.6. Pancreatic Lipase Inhibition

Ethanolic extracts of the flowers of *Spilanthes acmella* are demonstrated to inhibit pancreatic lipase activity (40% at 2 mg/mL concentration *in vitro*) [26]. The activity was compared with *Aframomum meleguetta* (90% inhibition) and proved to be inferior, whereas 0.75 mg/mL extract inhibited more pancreatic lipase than *Spilanthes*. 

### 4.7. Vasorelaxant (Effect on Blood Flow) and Antioxidant Activity [27, 28]

The plant extracts elicited vasorelaxations *via *partially endothelium induced nitric oxide and prostaglandin-I_2_ in a dose-dependent manner. However, the researchers suggested that other underlying mechanisms may participate. Significantly, the ethyl acetate extract exhibited immediate vasorelaxation in nanogram levels and is the most potent antioxidant in the diphenylpicryl hydrazine assay. The chloroform extract displays the highest vasorelaxation with the highest antioxidant concentration. Antioxidant potential of leaves of *Spilanthes acmella* was also studied recently by the researchers and they found that the potent antioxidant activity in the crude ethanol extract of the leaves of the plant was attributed to the presence of tannins, flavonoids and phenolic compounds.

### 4.8. Antimalarial and Larvicidal Effects

Spilanthol is more effective even at low doses against eggs and pupae. In pupae, it seems to work on nervous system as evident by abnormal movement like jerks, spinning and uncoordinated muscular activity. This suggested that the drug disturbed the nerve conduction somewhere. The mortality of pupae in short span of time upon exposure to the drug also indicated that spilanthol greatly disturbs the ongoing processes of histolysis and histogenesis. Many researchers also reported spilanthol as a potent larvicidal agent [13]. 

### 4.9. Aphrodisiac Action (Interaction with Testosterone and Sexuality) [1]

Aphrodisiac effect of the plant extract has been studied in male rats by Sharma et al., 2011. They stated that mount latency, intromission latency, ejaculation frequency, and postejaculatory interval were increased in a dose-dependent manner after oral administration of extract. Although exact quantification of these improvements was not given, estimation derived from graphs suggested that after 28 days of 150 mg/kg dose, the improvements were reduced in mount latency, intromission latency, and post ejaculatory latency. These benefits were more significant 28 days after supplementation relative to 14 days, suggesting a build-up effect. The plant proved to be superior to Viagra in all aspects studied except proerectile properties.

### 4.10. Antinociceptive Activity [29, 30]

Antinociceptive activity of the crude ethanol extract of *S. acemella *using acetic acid induced writhing model in mice is available elsewhere in literature. The animals of test groups received test substance at the dose of 250 and 500 mg/kg body weight. Positive control group was administered Diclofenac sodium (standard drug) at the dose of 25 mg/kg body weight, and vehicle control group was treated with 1% Tween 80 in water at the dose of 10 mL/kg body weight. Test samples, standard drug, and control vehicle were administered orally 30 min before intraperitoneal administration of 0.7% acetic acid. After an interval of 15 min, the mice were observed to be writhing (constriction of abdomen, turning of trunk, and extension of hind legs) for 5 min. Crude ethanol extract of *S. acemella *leaves was found to possess significant antinociceptive activity.

### 4.11. Immunomodulatory Activity [31]

Hexane and chloroform extracts of *Spilanthes acmella* were found to suppress nitric oxide production in stimulated macrophages at 80 mcg/mL by 72% and 85%, respectively. Isolated spilanthol demonstrated dose-dependent prevention of macrophage activation with 60% and 20% production of nitric oxide at 90 and 360 *μ*M concentrations, respectively. These inhibitory properties were accompanied by less nitric oxide synthetase and cyclooxygenase-2 mRNA and protein content, less cytokine production from macrophages, and less nF-kB activation in the nucleus.

### 4.12. Bioinsecticide and Convulsant Activity

The genus *Spilanthes *consists of 42 known species and several insecticidal compounds which have been reported in *Spilanthes mauritiana, S. alba, S. ocymifolia, S. oleracea, *and *Spilanthes acmella* [2, 12, 32]. Hexanic extract of *Spilanthes acmella *plant in rats was reported to induce full tonic-clonic convulsions accompanied by typical electrographic seizures in the electroencephalogram [25]. 

## 5. Toxicology

### 5.1. Evaluated on Zebrafish

It was found that plant contained a high yield of phytotestosterone [33]. Testosterone has an influence on growth rate and feed utilization in low dose-dependent variation in sheep and cattle [34]. Because the zebrafish embryo test is a highly sensitive toxicity test of chemical substances on animals, the result can be used as basic data for the toxicity test in higher animals and environmental contamination regulation. A study shows that *Spilanthes acmella* Murr. does not have any lethal effect on zebrafish embryo at 20% v/v, which was the highest concentration of the study, while significantly the lowest observable sublethal effect concentration was 10%. According to this study, crude extract of *Spilanthes acmella* Linn. Murr. can be used in animal feed at 0.01% v/v and 1% v/v, respectively, without any lethal, sublethal, and malformation effect [35]. 

### 5.2. Insecticidal Toxicity of Spilanthol

Extract of Spilanthol from the flower heads of *Spilanthes acmella* was found to be active against *P. xylostella *[36]. The extracts from *Spilanthes acmella *were most toxic against different mosquito species (i.e., *Anopheles*, *Culex, *and *Aedes*). The insecticidal property was attributed to spilanthol and alkamides. Besides, nonvolatile sesquiterpenoids and saponins were also reported [14, 32]. Ethanol extract of flower heads of *Spilanthes acmella* has shown a potent ovicidal, insecticidal, and pupacidal activity at dose of 7.5 ppm concentration with 100% of *Anopheles*, *Culex, *and *Aedes *mosquito [37]. The hexane extract of dried flower buds of *Spilanthes acmella* (3 N-isobutylamides: spilanthol, undeca-2E,7Z,9E-trienoic acid isobutylamide and undeca-2E-en-8,10-diynoic acid isobutylamide) was found active against *Aedes aegypti *larvae. Ethanolic extracts of *Spilanthes acmella* (whole plants) were screened against early 4th instar larvae of *Culex quinquefasciatus *[38]. Spilanthol was shown to be toxic against adults of *P. americana*. It is one of the most potent compound when compared with conventional insecticides such as carbaryl, lindane, and bioresmethrin with a potency found to be 1.3, 3.8, and 2.6 times, respectively [36]. 

## 6. Summary and Conclusion 

*Spilanthes acmella *is a well known plant in Indian traditional system of medicine with multiple pharmacological action and minor side effects. In this review, we concluded ethnobotany, phytochemistry, pharmacology, and toxicology in a descriptive manner. The summary of phytochemicals and pharmacological actions is tabulated in Tables 1 and 2, respectively. Extracts and phytoconstituents isolated from this plant have shown to produce different pharmacological response, which includes anticonvulsant, analgesic, anti-inflammatory, vasodilation, diuretic, and antimalarial effects. The most traditional use of this plant is to reduce toothache all over India as well as South America. Other traditional uses of *Spilanthes acmella* are as stomachic, stimulant, and antidiarrhoeal and is used rarely against tuberculosis. Many researchers proposed that whole plant has local anaesthetic, anti-inflammatory, antioxidant, aphrodisiac, antinociception, immunomodulator, and insecticidal effect. On the other hand, flower part has shown to produce diuretic, vasorelaxation, antifungal and pancreatic lipase inhibition properties. Its multiple traditional use and pharmacological responses allow us to write a review of *Spilanthes acmella. *This review will give all the scientific information in a concise manner to the scientific community. 

## Figures and Tables

**Table 1:** Chemical structures of secondary metabolites of *Spilanthes acmella*.

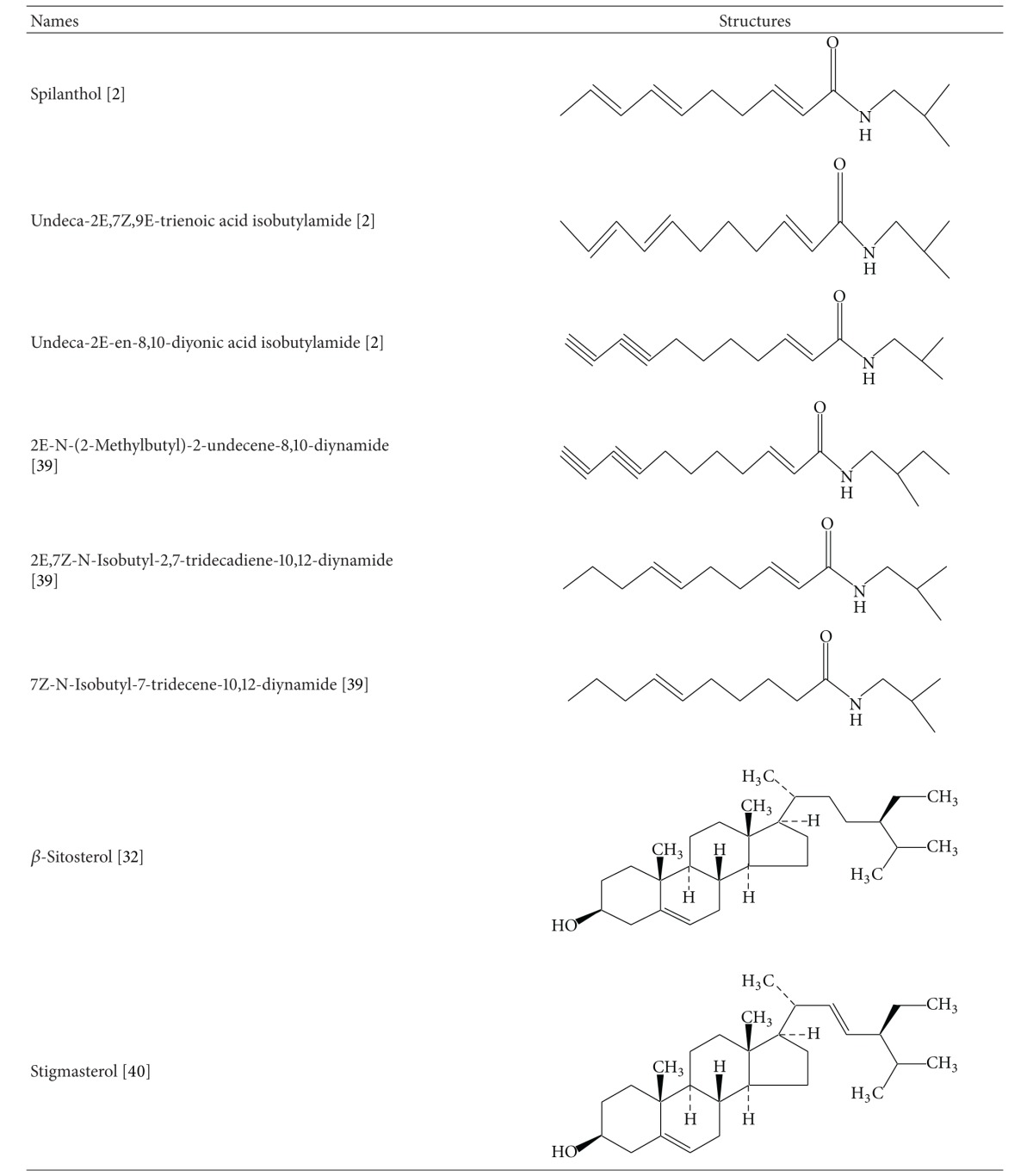 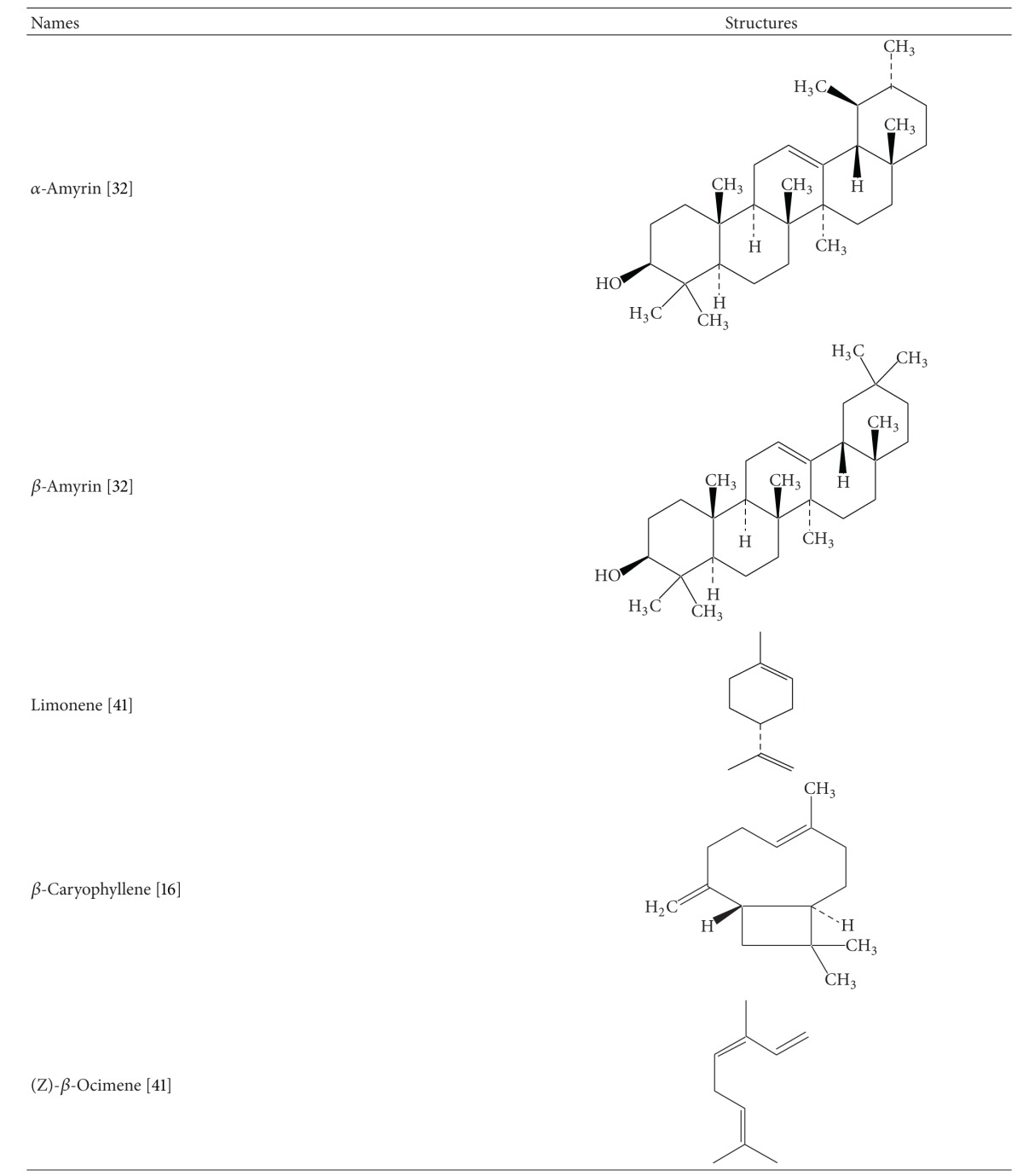 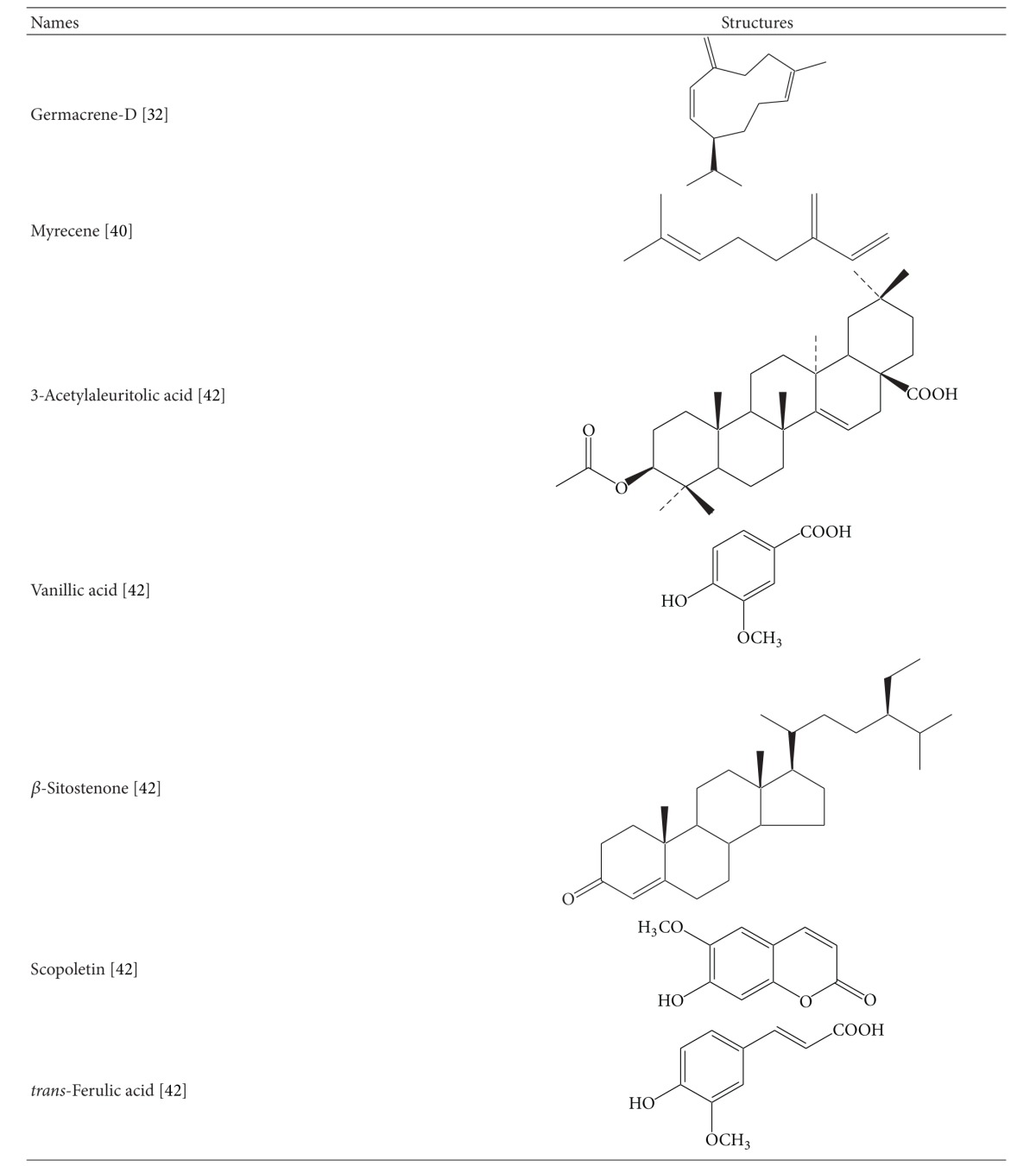 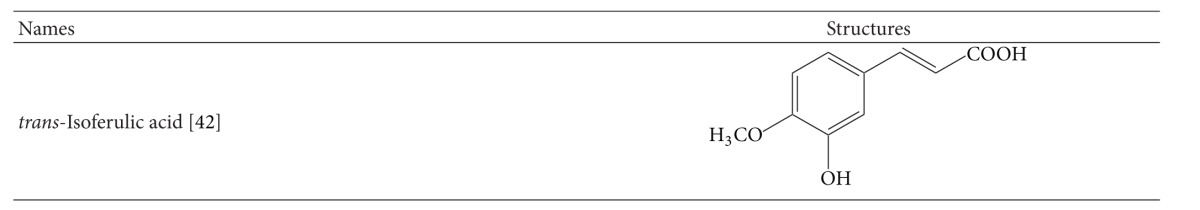

**Table 2:** Summary of pharmacological actions of *Spilanthes acmella. *

SL. no.	Pharmacological activity	Parts of plant used	Experimental models	Animals used	References
1	Local anaesthetic	Whole plant	Intracutaneous wheal in guinea pigs and plexus anaesthesia in frog	Guinea pig, frog	[19]
2	Antipyretic activity	Whole plant	Yeast induced pyrexia	Albino rats	[19]
3	Anti-inflammatory activity	Whole plant, leaves	Carrageenan induced paw oedema	Albino rats	[22, 31]
4	Analgesic activity	Whole plant	Tail flick method, acetic acid induced abdominal constriction	Albino rats	[22]
5	Antifungal activity	Flowers	—	—	[23]
6	Diuretic activity	Flowers (cold water extract), whole plant	Induction of dieresis using cold water extract	Albino rats	[24, 25]
7	Vasorelaxant activity	Flowers	Partially endothelium induced nitric oxide and PGI_2_	Albino rats	[30, 31]
8	Antioxidant activity	Leaves & whole plant	DPPH Assay, TBARs and SOD method	*Invitro*, no animal	[30, 31]
9	Pancreatic lipase inhibition	Flowers	—	*Invitro*, no animal	[29]
10	Antimalarial & larvicidal activity	Spilanthol extracted from whole plant	—	Eggs & pupae of vector	[37]
11	Aphrodisiac activity	Whole plant	—	Male rats	[1]
12	Antinociceptive activity	Whole plant	Acetic acid induced writhing	Mice	[31]
13	Immunomodulatory activity	Whole plant	—	Rats	[36]
14	Bioinsecticidal	Whole plant, leaves	—	—	[2, 12, 32]
15	Convulsant	Whole plant	Electroencephalogram (EEG) analysis	Albino rat	[25]
